# Beyond the Score Study: Retrospective Analysis of Single-Graft Kidney Transplant with Karpinski Score 4 Versus Score 5 Grafts

**DOI:** 10.3390/medicina61122074

**Published:** 2025-11-21

**Authors:** Matteo Zanchetta, Stefania Angela Piccioni, Giorgio Micheletti, Giuseppe Ietto, Vincenzo Li Marzi, Natale Calomino, Giulio Bagnacci, Andrea Collini, Guido Garosi, Gian Luigi Adani

**Affiliations:** 1 Surgical Oncology Unit, Department of Medicine Surgery and Neuroscience, University Hospital of Siena, 53100 Siena, Italy; 2 Kidney Transplant Unit, Department of Medicine Surgery and Neuroscience, University Hospital of Siena, 53100 Siena, Italy; 3 General, Emergency and Transplant Surgery Department, University Hospital of Varese, 21100 Varese, Italy; 4 Department of Medical, Surgical and Neurosciences, University of Siena, 53100 Siena, Italy; 5 Unit of Diagnostic Imaging, Department of Medical, Surgical and Neurosciences and of Radiological Sciences, University Hospital of Siena, 53100 Siena, Italy; 6 General Surgery Unit, Ospedale La Fratta, 52044 Cortona, Italy; 7 Nephrology, Dialysis and Transplantation Unit, Department of Medical Science, University Hospital of Siena, 53100 Siena, Italy

**Keywords:** kidney transplantation, graft, Karpinski score, extended criteria, marginal donor, older donor, end-stage renal disease, patient survival, graft survival

## Abstract

*Background and Objectives*: Considering the growing shortage of grafts available for kidney transplantation (KT) due to the increase in the number of end-stage renal disease patients, it is essential to utilize all transplantation options. The Karpinski score is a histological scoring system utilized for the evaluation of pre-implantation kidney biopsies from deceased donors. In contrast with Remuzzi Criteria, there has recently been a tendency to perform single KT using grafts with a Karpinski score of 4 or 5. This strategy allows two score 4 or 5 grafts to be used for two different recipients instead of a double-graft KT on one patient. The aim of this study was to analyze the outcomes of single-graft KT with a score of 4 versus 5, and to investigate possible correlations with the clinical characteristics of donors and recipients. *Materials and Methods*: Retrospective single-centre analysis of 100 KTs performed with a single Karpinski score 4 or 5 graft between January 2014 and December 2022. *Results*: Grafts with a Karpinski score of 5 harvested from donors older than 70 years of age had a statistically significant (*p* = 0.014) worse 5-year survival rate (50.0 +/− 18.6%) compared to younger donors (100% for score 5 grafts from donors aged 31–60, and 100% for score 5 grafts from donors aged 61–70). Conversely, donor’s age did not significantly affect the survival of score 4 grafts. *Conclusions*: The results suggest that a single-graft KT with a Karpinski score 5 graft may be a viable procedure with favourable outcomes. However, for Karpinski score 5 grafts, the use of an older donor beyond the age of 70 seems to be a significant negative factor for the long-term outcome. In such cases, a double KT would potentially be the optimal approach.

## 1. Introduction

For decades now, the first-line treatment for patients with end-stage renal disease (ESRD) has been kidney transplantation (KT) [1,2]. However, at a time when there is a growing shortage of organs available for KT due to the increase in the number of ESRD patients [3] and longer life expectancy, the widening gap between available donors and patients on the waiting list is becoming increasingly difficult to bridge [4]. In order to increase the number of available organs, it is essential to raise awareness of the importance of donation and to utilize all available transplantation options to minimize the discard rate and maximize the number of grafts used, regardless of anatomical variants that may potentially increase the risk of peri-operative complications or donor advanced age or comorbidities [5]. The concept of the “marginal donor” was first introduced at the 2001 Crystal City meeting [6]. The pool of potential donors was expanded by the addition of new “extended criteria”, which allowed some individuals who were previously excluded due to age or specific comorbidities to donate. These donors have been defined as “Extended Criteria Donors” (ECD). The technique of dual kidney transplantation (DKT) is related to the concept of the “marginal donor” and further widens the availability of grafts. Deceased donors with limited renal functional capacity represent a notable proportion of potential kidneys that are destined to be discarded or non-recovered. The physiological functional decrease and potential comorbidities of older donors may compromise a priori the graft viability as a single, and therefore a DKT has been found to be a means to overcome this deficit when transplanting marginal grafts, since two suboptimal grafts working together may still be enough to sustain a single recipient. Furthermore, several centres have raised the age limit for deceased donors due to increasing life expectancy in the general population [7]. In Italy, a pre-transplant biopsy is required when considering a kidney from a “suboptimal” ECD. The criteria of Remuzzi [8], based on the Karpinski score [9], dictate that kidneys with a score lower than 4 are transplanted individually, those with a score from 4 to 6 are transplanted in pairs, and those with a score of 7 or higher are discarded. The Karpinski score is a histological scoring system utilized for the evaluation of pre-implantation kidney biopsies from deceased donors. The primary purpose of this evaluation is to ascertain the presence of chronic damage, including glomerulosclerosis, interstitial fibrosis, tubular atrophy, and vascular changes [9]. The results of this evaluation assist in determining the suitability of the organ for transplantation. Single-organ KT from “marginal” grafts is considered and performed primarily to expand the limited donor organ pool and reduce the high morbidity and mortality associated with long-term dialysis. Therefore, over time there has been a tendency to use single grafts that score 4 or 5, deviating from the Remuzzi criteria. This strategy allows two Score 4 or 5 kidneys to be used for two different recipients instead of a DKT in one patient. This expands the pool of available kidneys for transplantation and helps to fulfil more requests. However, it is important to evaluate the hypothesis of a more flexible and comprehensive approach through the analysis of long-term outcomes of such KTs. Therefore, the aim of this study was to analyze the outcomes of single-graft KT with a score of 4 versus 5 performed at our Institution, and to investigate potential correlations with the clinical characteristics of donors and recipients, hypothesizing that the age of the donors of such suboptimal grafts may have an influence on the long-term outcomes, especially on the survival of the graft itself.

## 2. Materials and Methods

**Figure 1:**
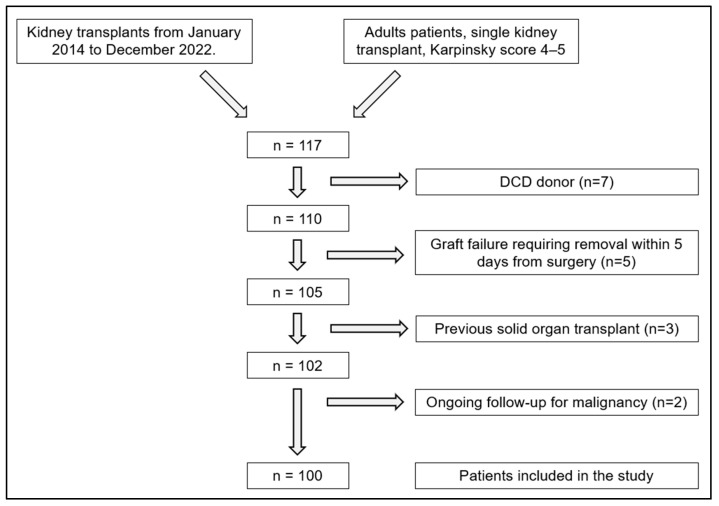
Flowchart explaining the selection of patients included.

This is a retrospective study analyzing data from 100 patients who underwent single KT with a graft with a Karpinski score of 4 or 5 between January 2014 and December 2022 at the Department of General Surgery and Kidney Transplantation of the Azienda Ospedaliero-universitaria Senese. The inclusion criteria necessitated that patients were adults who had received a deceased donor graft with a Karpinski score of 4 or 5 upon biopsy, and had undergone a single-kidney transplantation. Exclusion criteria for this study included transplants with grafts from donors after cardiac death (DCD) (n = 7), graft failure requiring removal for any reason (e.g., thrombosis of the vein or artery) within five days of the procedure (n = 5), a history of solid organ transplants (n = 3), and ongoing follow-up (FU) for past malignancy (n = 2). The selection criteria used in the study are shown in Figure 1.

Delayed Graft Function (DGF) was defined as the need for dialysis during the first week after transplantation. Graft survival was calculated from the time of transplantation until the point of cessation of function or explanation. The moment the patient permanently returned to dialysis was defined as cessation of function. Explanation beyond the fifth postoperative day was performed for acute postoperative vascular complications, bleeding, and infection. Cold ischemia was defined as the interval from initiation of donor in vivo cold organ preservation to removal of the graft from cold storage. Warm ischemia time was defined as the interval from the removal of the graft from cold storage to the establishment of reperfusion with warm blood, inclusive of surgical anastomosis time. All patients provided written informed consent to collect and use their clinical data in our institutional database.

### 2.1. Donors

Donor characteristics were obtained from the Integrated Transplant Management System (eGIT) of Regione Toscana in Italy, the official secured database collecting all the information pertaining to the transplantations occurring within the Regione Toscana. The data gathered included age at donation, body mass index (BMI), comorbidities (e.g., hypertension, cardiomyopathy, vasculopathy, diabetes, hepatopathy, pneumopathy, psychiatric pathologies, other comorbidities), length of stay in the intensive care unit (ICU), and use of inotropes. All donors had a serum creatinine level below 2 mg/dL at the time of donation.

### 2.2. Graft

Data on transplanted kidney grafts were collected from accessible documents within the eGIT system. The grafts and KTs chosen for our study were classified according to the pre-implantation Karpinski score of 4 or 5. Both warm (WIT) and cold (CIT) ischemia times were collected. Kidney biopsies are performed by the surgeon after organ removal. Tissue samples are carefully taken from the lower pole of the kidney using an 11-blade scalpel, with a focus on targeting the cortical tissue. The specimens are sent to the pathology department of the transplant centre for thorough analysis. A minimum of 20 glomeruli is considered essential to ensure sample adequacy. The histopathological evaluation involves a thorough assessment of the glomerular, interstitial, tubular, and vascular compartments using the Karpinski scoring system. Each compartment is graded on a scale from 0 (no chronic changes) to 3 (severe chronic changes), resulting in a total score ranging from 0 to 12. Notably, this scoring system bears substantial resemblance to the Remuzzi (or Pirani) score, differing only in minor definitions of vasculopathy and the number of glomeruli required for validity.

### 2.3. Imaging

All donors underwent imaging studies prior to organ removal as part of the preoperative evaluation. Donors underwent ultrasound (US) scans to assess key parameters such as kidney size and cortical thickness, and to identify potential abnormalities of the renal parenchyma and excretory tract. All recipients underwent a post-operative FU with US to monitor outcomes and assess graft function.

### 2.4. Recipients

Recipient data were obtained from a continuously updated transplant database, supplemented by information obtained at our centre during post-transplant FU. Follow-up visits took place at one, three, six and twelve months after KT, and annually thereafter. The data gathered included age at transplantation, dialysis modality (hemodialysis [HD] or peritoneal dialysis [PD]), BMI, comorbidities (e.g., hypertension, diabetes, cardiopathy, other comorbidities), and DGF.

### 2.5. Statistical Analysis

Statistical analysis was performed using SPSS Statistics Version 26. Continuous variables were explored visually and with the Shapiro–Wilk test; between-group comparisons used Student’s *t* test or Mann–Whitney U otherwise. Categorical variables were compared using χ^2^ or Fisher’s exact test, as appropriate. Graft and patient survival were estimated with Kaplan–Meier and compared using the log-rank test. Results are reported as *p*-values, with a threshold of *p* < 0.05 considered statistically significant.

## 3. Results

A total of 100 patients (63 men and 37 women) were included in the study. Clinicopathological characteristics of the donors, histopathological characteristics of the grafts, and clinicopathological characteristics of recipients are shown in Table 1, Table 2 and Table 3, respectively.

Sixty-seven grafts had a Karpinski score of 4 and thirty-three had a Karpinski score of 5. The donors’ population was divided into two groups according to whether grafts had a Karpinski score 4 or 5 (Table 4). There were no statistically significant differences observed when examining donors divided by the Karpinski score of their graft.

Statistically significant differences were observed in the vascular score (*p* < 0.001), and glomerular score (*p* = 0.027) of the grafts divided by their Karpinski score of 4 or 5 (Table 5).

Karpinski 5 grafts had a statistically significant higher vascular score, with 12.12%, 84.85%, and 3.03% of them having 1, 2, and 3 points, respectively. Similarly, grafts with a Karpinski score 5 had a statistically significant higher glomerular evaluation, scoring 0, 1, 2, and 3 points in 9.09, 81.82%, 9.09%, and 0% of the cases, respectively. No significant differences were found for size, cortical thickness, or parenchymal and excretory tract abnormalities. There were no statistically significant differences identified among the recipients, divided according to the Karpinski score of the graft they received, with respect to their medical history and comorbidities (Table 6).

**Figure 2:**
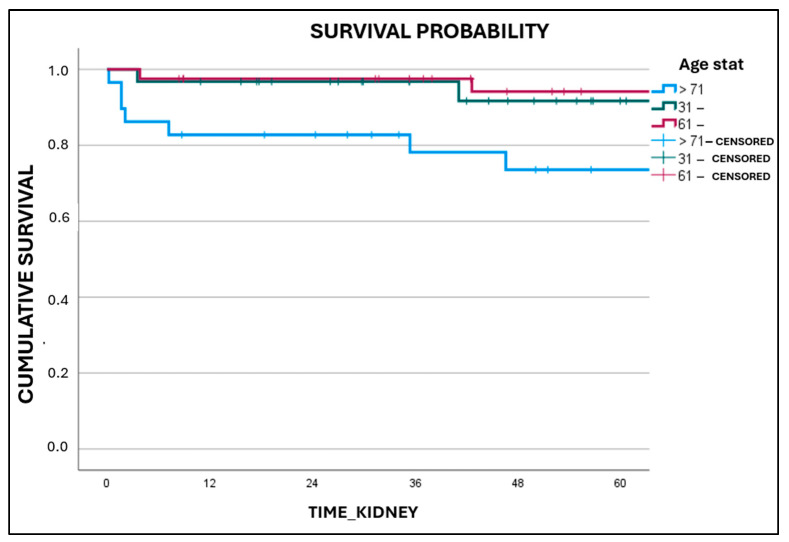
Graft survival related to donor age (*p* = 0.010).

Similarly, no significant variations were observed in the occurrence and duration of DGF, cessation of function, and mortality. The 5-year survival rate for recipients of Karpinski score 4 grafts was 96.5% ± 2.5 Standard Error (SE), while recipients of Karpinski score 5 grafts had a survival rate of 93.8% ± 4.2 SE. The survival curves did not show a statistically significant difference (*p* = 0.987) based on the log-rank Mantel–Cox test (Supplementary Materials Figure S1). Grafts with a Karpinski score of 4 had a 5-year survival rate of 87.9% ± 4.4 SE, while grafts with a Karpinski score of 5 had a 5-year survival rate of 86.6% ± 6.4 SE. The log-rank Mantel–Cox test showed no significant difference between the survival curves (*p* = 0.857) (Supplementary Materials Figure S2). A study was conducted to evaluate graft survival based on the age of the donor. The study used three age groups for the donors: 31–60, 61–70, and 71 years or older. The results showed a statistically significant decrease in 5-year graft survival for donors aged 71 years or older (*p* = 0.010) (Figure 2).

**Figure 3:**
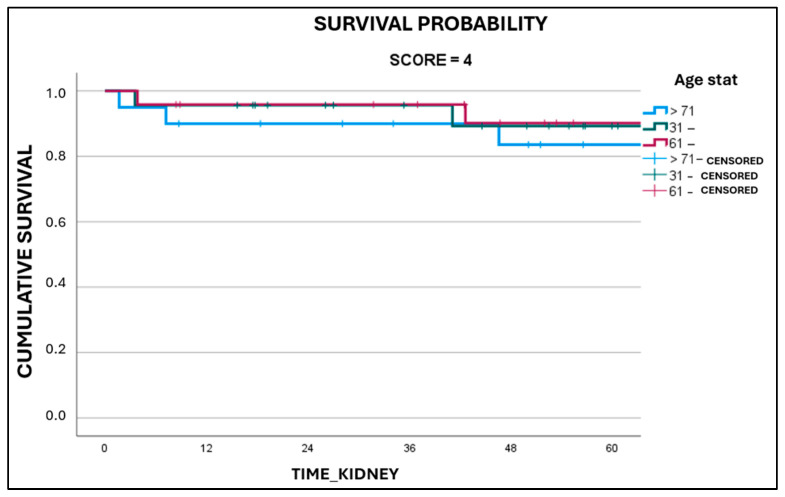
Graft survival as a function of donor’s age in Karpinski 4 grafts.

**Figure 4:**
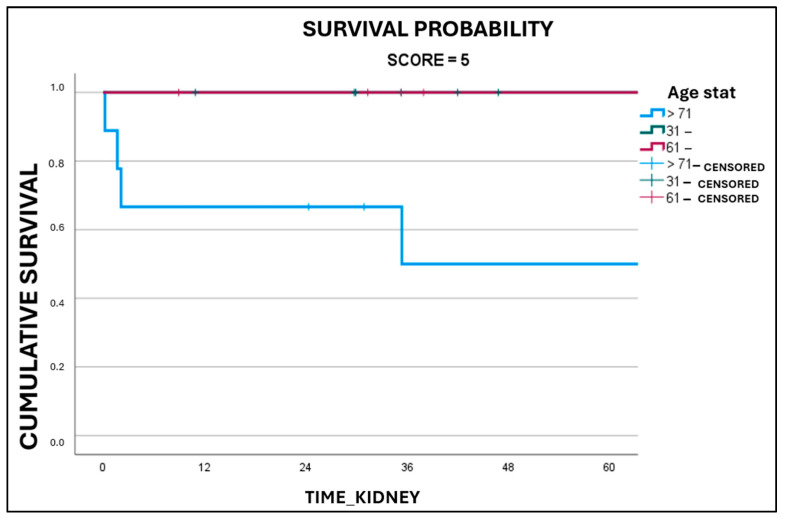
Graft survival as a function of donor’s age in Karpinski 5 grafts (*p* = 0.014).

Upon analyzing separately grafts with Karpinski scores of 4 (Figure 3) and 5 (Figure 4) according to their respective donors’ age, it was discovered that grafts with a Karpinski score of 5, harvested from donors older than 70 years of age, had a statistically significant worse survival (*p* = 0.014).

For Karpinski score 5 grafts from donors older than 70 years of age, the 5-year survival rate (50.0 +/− 18.6%) was significantly lower compared to the other age groups (100% for score 5 grafts from donors aged 31–60, and 100% for score 5 grafts from donors aged 61–70). The 5-year survival rates for Karpinski score 4 grafts from donors older than 70 years of age, between 61 and 70 years, and between 31 and 60 years of age, were 75.2 +/− 11.2%, 90.2 +/− 6.7%, and 89.3 +/− 7.3%, respectively. However, there were no significant differences observed in the length of stay in ICU (*p* = 0.712) or in the utilization of inotropes (*p* = 0.441).

## 4. Discussion

In order to increase the pool of available organs, it is essential to use all transplant options, minimize the discard rate and maximize the number of grafts used. Despite the increased utilization of grafts from older donors observed in recent years, this practice is not without its challenges. The physiological functional decrease with ageing and the potential comorbidities of such older donors may compromise a priori the graft viability as single in the recipient, and therefore a DKT has been found to be a means to overcome this deficit when transplanting marginal grafts. The present study provides data on which to elaborate on a daily clinical dilemma experienced by transplant surgeons when deciding whether to use a marginal graft or not. Kidney transplantation with a living donor is a feasible alternative, offering shorter wait times and better short- and long-term outcomes for their recipients compared to deceased donors [9,10]. Dual kidney transplantation is a valuable option for reducing the number of patients on the waiting list and avoiding the discarding of organs from suboptimal donors. Simultaneously transplanting two suboptimal kidneys to the same recipient may be more effective than transplanting a single kidney, thereby overcoming the imbalance between the nephron mass of so-called “marginal donors” and the metabolic needs of the recipient [11,12]. This approach avoids discarding organs that are unsuitable for individual transplantation, offering satisfactory and valid results [13,14]. As both the recipient and potential donor populations age, several centres have raised the age limit for deceased and living donors [7,15,16,17,18,19]. Although there is a higher incidence of graft loss at one and three years and higher mortality at one year, the scientific community has accepted the use of donors in their 70s. This is because it has been shown to nonetheless reduce recipients’ mortality compared to those ESRD patients who remain on dialysis [16]. Therefore, kidney donation from older donors is likely to play an ever-increasing role in the future. Considering grafts from ECD, although they have a higher incidence of DGF, primary non-function (PNF), and a lower graft-survival rate, their utilization has greatly expanded the number of available organs [20,21]. Furthermore, recipients of ECD grafts still have lower mortality rates compared to those who continue with dialysis [19,20]. Several studies have suggested that the pretransplant histology score has a limited role due to its focal representation of the subcapsular area of the kidney, and its interpretation, which is operator dependent when using the Karpinski score [22,23]. On the other hand, many other authors advocate for pre-transplantation histological evaluation [8,24,25,26]. In Italy, the use of grafts from ECD and elderly donors requires a pre-transplant kidney biopsy to be performed by law to evaluate the feasibility of the KT. Higher glomerular and vascular scores associate with a worse outcome in terms of development of DGF, graft function and graft survival [9] compared to lower scores. The severity of vascular pathologies is considered a major determinant of KT outcome [25,27], but it is difficult to predict the severity of vascular pathology in a marginal donor without biopsy, as it does not necessarily correlate with age. As individuals age, their renal function progressively declines due to nephron loss. This results in a reduction in the glomerular filtration rate, estimated to decrease by approximately 1 mL/min per year from the age of 40 onwards [28]. Similarly, a long-standing arterial hypertension correlates with worse vascular score. Examining our study population, Karpinski score 4 grafts were significantly better than score 5 grafts regarding glomerulosclerosis and arteriosclerosis (*p* < 0.05). Confronting donors divided according to the Karpinski score of their graft, there were no statistically significant differences neither in age nor in the incidence of arterial hypertension. No statistically significant differences were observed confronting the recipients divided according to the Karpinski score of the graft they received. Our analysis revealed a significant decrease in graft survival when the donor was 71 years of age or older. While the scientific community generally accepts the use of donors in their 70s for KT, it is important to note that older donors may have a significant number of comorbidities that can compromise the functionality of the graft from the beginning. For this reason, it is necessary to perform a pre-transplant biopsy to assess the overall condition of the potential graft. Based on the Remuzzi criteria, grafts with a Karpinski score ranging from 4 to 6 should be transplanted in pairs to overcome their suboptimal status. However, in recent years, many centres have opted to use grafts with a score of 4 or 5 for single transplantation, contrary to Remuzzi’s classical indications. Some authors have reported that patients who received a double transplant and subsequently underwent explantation of one of the two grafts exhibited acceptable renal function in the long-term (between five and ten years) [15,29]. For years, the Department of General Surgery and Renal Transplantation at Azienda ospedaliero-universitaria Senese has been performing single score 5 transplants. Our study analyzed short- and long-term outcomes of score 4 versus score 5 single grafts. Despite worst glomerular and vascular scores for Karpinski 5 grafts, the results showed a slight negative trend in graft survival compared to Karpinski 4 grafts, but this was not statistically significant. Similarly, the analysis of patient survival after transplantation revealed no statistically significant difference between the two populations. This confirms the viability of single transplantation for Score 5 grafts. However, we noticed a significant decrease in graft survival for Karpinski score 5 grafts harvested from donors older than 70 years of age. Considering the absence of statistically relevant differences in donor and recipient populations, what emerges from our analysis is that the age of the donor, specifically 71 years or older, appears to be a negative key factor in graft survival when transplanting a single Karpinski score 5 kidneys.

Our study has some limitations, such as the retrospective nature and the single-centre population. The relatively small cohort diminishes the statistical power of the analyses. Consequently, the limited number of events restricts the use of a Cox multivariate analysis (event per borderline variable), increasing imprecision and the risk of overfitting; larger cohorts, possibly multicenter, would be needed.

Nevertheless, the topic remains worthy of further exploration. It is recommended that future research be conducted by means of evaluating larger cohorts in a multi-centre analysis. This would allow for the evaluation of a greater number of participants and, moreover, serve to strengthen the statistical power of the results. Moreover, the impact of pre-implant mechanical perfusion, which has been shown to reduce the incidence of DGF, enhance survival, and promote regeneration of marginal grafts [30], may prove particularly advantageous in the context of high-risk grafts derived from older donors.

## 5. Conclusions

In the modern era of solid organ transplantation, the disparity between the demand for transplants and the supply of organs is difficult to overcome. To expand the pool of available organs, it is essential to increase the number of donations through awareness programmes and utilize all transplantation options. In recent years, many centres have increased the accepted age of donors. Considering potential comorbidities affecting the graft functionality, a pretransplant biopsy is required for older donors. We retrospectively evaluated the outcomes of 100 KTs performed with single Karpinski score 4 or 5 grafts in our Institution. The results of this study suggest that a single kidney transplant with a Karpinski score 5 graft is a feasible procedure with acceptable results. However, for Karpinski score 5 grafts, an older donor beyond the age of 70 appears to be a significant negative factor for the long-term outcome. This indicates that a DKT with such grafts would be the optimal approach.

## Figures and Tables

**Table 1:** Clinicopathological characteristics of donors.

**Average age (years)**	65 ± 12 (31–80)	
**Average BMI (kg/m^2^)**	25.9 ± 5.0 (17.1–51.1)	
**ICU length of stay (days)**	2 ± 4 (1–19)	
	*Present*	*Absent*
**Use of inotropes**	72	28
**Hypertension**	45	55
**Cardiomyopathy**	16	84
**Vasculopathy**	20	80
**Dyslipidemia**	26	74
**Diabetes mellitus**	8	92
**Hepatopathy**	0	100
**Pneumopathy**	10	90
**Psychiatric pathology**	21	79

**Table 2:** Histopathological characteristics of the grafts.

**Karpinski score 4**	67			
**Karpinski score 5**	33			
**Size (mm)**	110.0 ± 15 (80–130)			
**Cortical thickness (mm)**	15.0 ± 3.4 (9.0–25.0)			
				
	0	1	2	3
**Vascular score**	0	57	42	1
**Interstitial score**	3	93	4	0
**Tubular score**	2	97	1	0
**Glomerular score**	15	82	3	0

**Table 3:** Clinicopathological characteristics of recipients.

**Average age at KT (years)**	57 ± 11 (25–71)	
**Average CIT (hours)**	15 ± 4.9 (6–26.5)	
**Average WIT (minutes)**	30.0 ± 5 (14–43)	
**Average BMI (kg/m^2^)**	25.2 ± 5 (16–34)	
**Hemodyalisis (#)**	75	
**Peritoneal dyalisis (#)**	19	
		
	Present	Absent
**Diabetes mellitus**	19	81
**Hypertension**	66	34
**Cardiomyopathy**	21	79
**DGF**	52	48

**Table 4:** Characteristics of donors divided in two groups according to the Karpinski score of their graft.

DONORS	K SCORE 4 N = 67	K SCORE 5 N = 33	*p*
**Age**			0.435
31–60	23	8	
61–70	24	16	
≥71	20	9	
**BMI**			0.092
<18.5	1	0	
18.5–24.9	30	10	
25–29.9	26	17	
>30	10	6	
**ICU length of stay (days)**			0.066
1	26	9	
2–3	21	9	
4–7	16	7	
>7	4	8	
**Inotropes use in ICU**			0.404
Yes	50	22	
No	17	11	
**Arterial hypertension**			0.076
Yes	26	19	
No	41	14	
**Cardiopathy**			0.115
Yes	8	8	
No	59	25	
**Vasculopathy**			0.395
Yes	15	5	
No	52	28	
**Dyslipidemia**			0.211
Yes	20	6	
No	47	27	
**Diabetes mellitus**			0.064
Yes	3	5	
No	64	28	
**Hepatopathy**			/
Yes	0	0	
No	67	33	
**Pneumopathy**			0.357
Yes	8	2	
No	59	31	
**Psychiatric pathology**			0.971
Yes	14	7	
No	53	26	

**Table 5:** Anatomical and histological characteristics of the grafts.

GRAFT	K SCORE 4 n = 67	K SCORE 5 n = 33	*p*
**Size (mm)**	109.5 ± 9.4	112.6 ± 10.8	0.149
**Cortical thickness (mm)**	14.9 ± 3.3	15.1 ± 2.7	0.827
**Side of harvesting**			**0.026**
Right kidney	34	9	
Left kidney	33	24	
**Vascular score**			**0.001**
1	53	4	
2	14	28	
3	0	1	
**Interstitial score**			0.189
0	2	1	
1	64	29	
2	1	3	
3	0	0	
**Tubular score**			0.221
0	2	0	
1	65	32	
2	0	1	
3	0	0	
**Glomerular score**			**0.027**
0	12	3	
1	55	27	
2	0	3	
3	0	0	

**Table 6:** Demographic and clinical variables of the recipients.

RECIPIENTS	K Score 4 N = 67	K Score 5 N = 33	*p* Value
**Age at transplant**			0.321
>30	1	0	
31–60	45	18	
61–70	21	15	
**BMI**			0.118
18.5–24.9	27	19	
25.0–29.9	33	10	
>30	7	4	
**Cold ischemia**	15.2 ± 3.8	15.7 ± 3.2	0.503
**Warm ischemia**	32.8 ± 4.8	32.3 ± 5.1	0.651
**Dialysis**			0.557
**Hemodialysis**	50	25	
**Peritoneal dialysis**	14	5	
**Pre-emptive**	3	3	
**Diabetes mellitus**			0.139
Yes	10	9	
No	57	24	
**Arterial hypertension**			0.424
Yes	46	20	
No	21	13	
**Cardiopathy**			0.627
Yes	15	6	
No	52	27	
**DGF**			0.946
Yes	35	17	
No	32	16	
**5-year graft survival**			0.979
Yes	8	4	
No	59	29	
**5-year patient survival with functioning graft**			0.986
Yes	4	2	
No	63	31	

## Data Availability

Data are available on considerate request.

## Supplementary Materials

The following supporting information can be downloaded at: https://www.mdpi.com/article/10.3390/medicina61122074/s1.
