# Supplementary material for: Beyond the Score Study: Retrospective Analysis of Single-Graft Kidney Transplant with Karpinski Score 4 Versus Score 5 Grafts
Source: Medicina (Kaunas). 2025 Nov 21;61(12):2074. doi: 10.3390/medicina61122074 (PMC12734436; doi:10.3390/medicina61122074)
Supplement: Supplementary file 1 [file medicina-61-02074-s001.zip › medicina-3956449-supplementary.pdf]

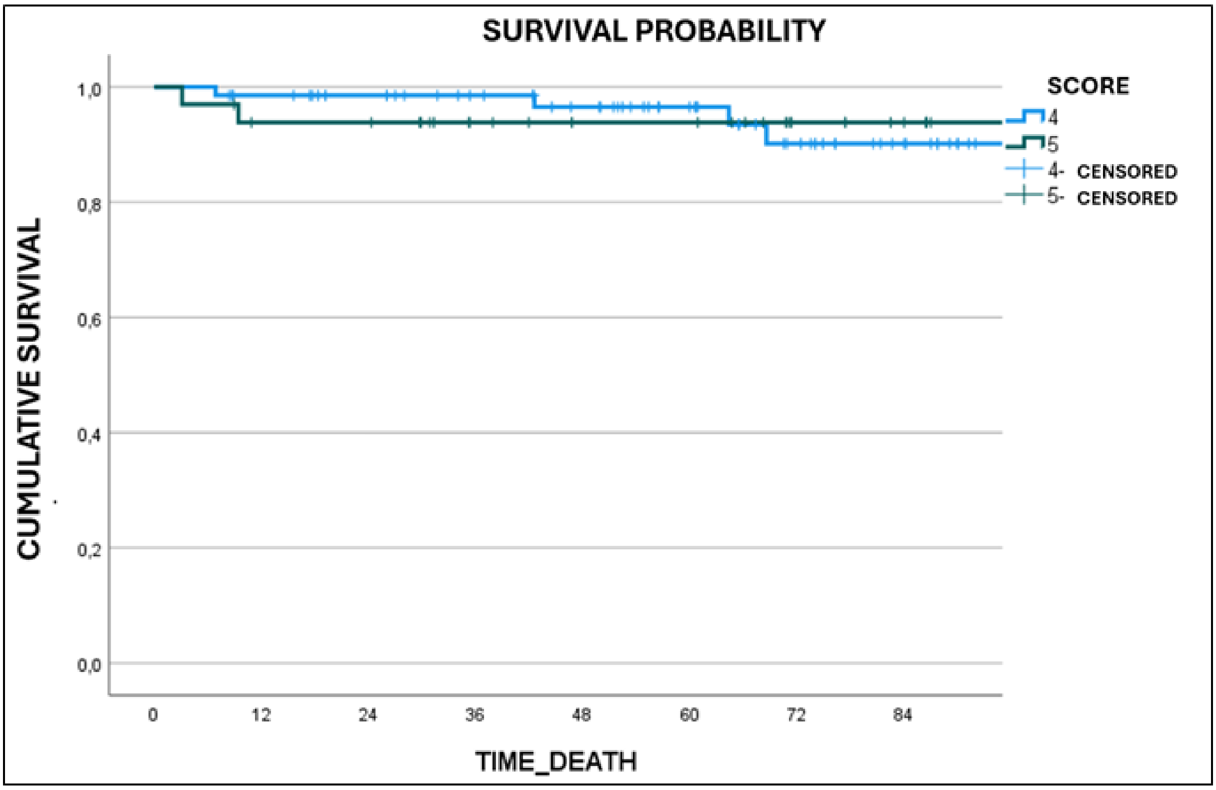

Figure S1. Five-year survival of recipients in Karpinski 4 group vs recipients in Karpinski score 5 group (p = 0.987).

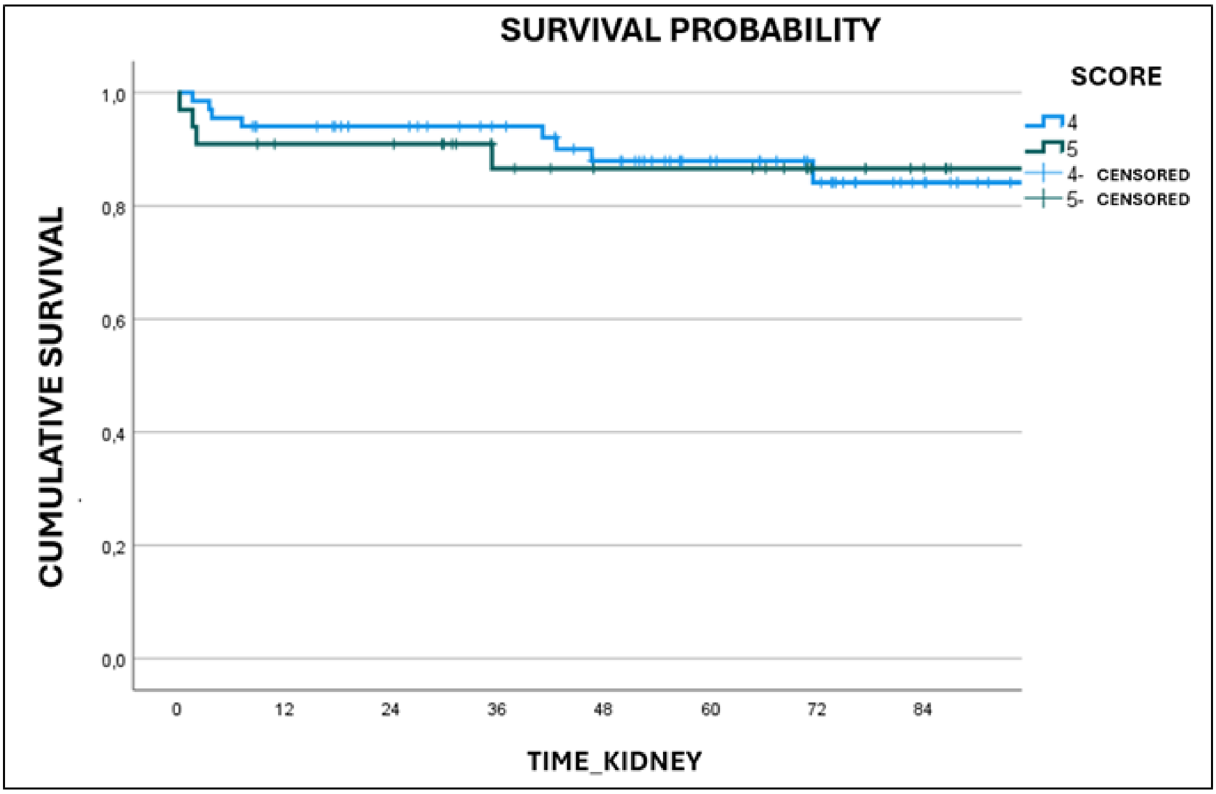

Figure S2. Five-year survival of grafts with a Karpinski score 4 vs grafts with a Karpinski score 5 (p = 0.712).
